# Toward GPGPU accelerated human electromechanical cardiac simulations

**DOI:** 10.1002/cnm.2593

**Published:** 2013-09-20

**Authors:** Guillermo Vigueras, Ishani Roy, Andrew Cookson, Jack Lee, Nicolas Smith, David Nordsletten

**Affiliations:** Department of Biomedical Engineering, King's College LondonUK

**Keywords:** GPU, cardiac electrophysiology, tissue mechanics, electromechanics

## Abstract

In this paper, we look at the acceleration of weakly coupled electromechanics using the graphics processing unit (GPU). Specifically, we port to the GPU a number of components of 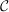
Heart—a CPU-based finite element code developed for simulating multi-physics problems. On the basis of a criterion of computational cost, we implemented on the GPU the ODE and PDE solution steps for the electrophysiology problem and the Jacobian and residual evaluation for the mechanics problem. Performance of the GPU implementation is then compared with single core CPU (SC) execution as well as multi-core CPU (MC) computations with equivalent theoretical performance. Results show that for a human scale left ventricle mesh, GPU acceleration of the electrophysiology problem provided speedups of 164 × compared with SC and 5.5 times compared with MC for the solution of the ODE model. Speedup of up to 72 × compared with SC and 2.6 × compared with MC was also observed for the PDE solve. Using the same human geometry, the GPU implementation of mechanics residual/Jacobian computation provided speedups of up to 44 × compared with SC and 2.0 × compared with MC. © 2013 The Authors. *International Journal for Numerical Methods in Biomedical Engineering* published by John Wiley & Sons, Ltd.

## 1. INTRODUCTION

The ability to predict the electromechanical behavior of the heart from imaging and other physiological data is one of the compelling, yet still only partially fulfilled, goals of the personalized healthcare [1–3]. The challenge that is central to bringing electromechanical modeling into the clinic is the process of patient-specific tailoring of the model as well as *in silico* treatment evaluation, both of which are processes requiring many electromechanical simulations. Patient-specific tailoring of models requires coupling patient data and model parameters using parameter estimation algorithms, which require the iterative solution of the model for a varied range of parameters [4, 5]. With a parameterized model, numerous simulations may be run to examine different potential treatment strategies. Although a number of authors have developed effective models and tools for simulating electromechanics, their use in diagnosis or treatment planning requires model analysis to conclude in clinically relevant time-scales, mandating continued improvement of simulation technologies.

The GPU architecture is a highly promising hardware with significant potential to accelerate cardiac electromechanics simulations. Toward this goal, a number of previous studies have already investigated the acceleration of the electrophysiology problem through GPUs [6–10]. Building on this work, in this paper, we propose the acceleration of the human scale electrical activation simulation and a novel GPU-based implementation of cardiac mechanics, which constitutes the first implementation of *weakly coupled* electromechanics on this platform. We analyze these parallel implementations by quantifying the computational gain of function, show the potential of this technology, to broaden the application of these types of Virtual Physiological Human (VPH) models.

The rest of the paper is organized as follows. In Section 2, previous studies of electrophysiology and electromechanics problems are reviewed. In order to understand architectural and programmability aspects of the GPU, Section 3 analyzes the main features of this parallel platform. Section 4 describes electrophysiology and mechanics models and numerical methods used in our CPU and GPU implementations. Section 5 presents CPU and GPU implementations for accelerating cardiac electromechanics simulations. A performance comparison between CPU and GPU versions is shown in Section 6, and their results are discussed in the conclusion (Section 7).

## 2. RELATED WORK

In order to tackle the computational barrier to the clinical translation of cardiac human models, some approaches have already been proposed that exploit parallel clusters facilities for simulating electrical activity [11, 12]. Although these works propose efficient High Performance Computing (HPC) implementations, the use of such large-scale computational facilities results in high cost in terms of price and power consumption and is less accessible in most clinical environments.

As the GPU has emerged as an efficient platform providing a good power/performance ratio, a number of groups have investigated the use of GPUs for accelerating cardiac electrophysiology simulations. Bartocci *et al*. [6] have proposed the implementation of the ODE solver on the GPU and evaluated the approach using 2D tissues. Another approach introduced by Vigmond *et al*. [7] has aimed at facilitating the acceleration of the ODE solver through the application of GPUs, demonstrating its efficacy in small mammalian hearts. A further extension has been recently proposed by Rocha *et al*. [8], who used the single-precision GPU to solve the system of PDEs and ODEs present in the Monodomain model to solve 2D tissue simulations. Plank *et al*. [9] recently developed a solution proposing a multi-GPU implementation for performing cardiac simulations using a rabbit model, showing significant speedups with respect to their parallel CPU code CARP.

Building on these efforts, in this paper, we look to simulate both electrophysiology and mechanics on the GPU. Although some recent works have simulated mechanics using low-order refined meshes [13, 14], most mechanical models of the heart use incompressible quasi-static finite elasticity solved on high-order curvilinear hexahedral elements [15–18]. Beyond the change in interpolation scheme, the inherent nonlinearity of cardiac mechanics and structure of the linearized system poses significantly different challenges to those faced with parallelization of electrophysiology. In this paper, we focus on the initial acceleration of mechanics computations by porting algorithms for Jacobian matrix and residual evaluations. In this context, we show the benefits provided by GPUs for simulating both the electrical activity and mechanical deformation in the human heart.

## 3. GPU ARCHITECTURE DESCRIPTION

The appearance of Compute Unified Device Architecture (CUDA) [19] has enabled the use of GPUs as powerful computing platforms and enabled their recent extension to general-purpose computing. The CUDA model is a hardware and software architecture to perform computations on the GPU as a data-parallel computing device, without the need of using a graphics API [19].

**Figure 1:**
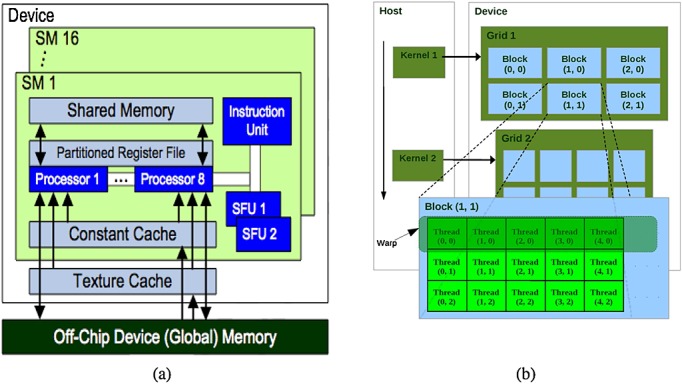
(a) Compute Unified Device Architecture (CUDA) hardware interface for the Nvidia GPU G80 (b) CUDA programming model [19].

Figure 1 illustrates the hardware interface of CUDA for the Nvidia GPU G80. This parallel single instruction multiple data (SIMD) architecture is endowed with up to 128 cores, where thousands of threads run in parallel. These cores are organized into 16 multiprocessors (SMs), each one having a set of 32-bit registers, constants and texture caches, and 16 KB of on-chip shared memory as fast as local registers (one cycle latency). At any given cycle, each core executes the same instruction on different data (SIMD), and communication between multiprocessors is performed through global memory.

Figure 1 outlines the CUDA programming model. CUDA consists of a set of C language library functions, which the programmer uses to specify the structure of a CUDA program. A CUDA program consists of two subprograms as follows: the CPU part (*host subprogram*) and the GPU part (*device subprogram*). The host subprogram prepares the GPU execution, moving data from CPU main memory to the GPU memory. Also, the host subprogram is in charge of setting up all the parameters involved in the execution and launching the device subprogram. In its turn, the device code is organized in functions or *kernels*. Each kernel is executed in parallel by each GPU thread.

A kernel execution is decomposed into blocks that run logically in parallel (physically if there are resources available on the GPU). Assembled by the developer, a block consists of a group of threads that is mapped to a single multiprocessor, where threads can share up to 16 KB of memory and also synchronize through barrier primitives. However, communication among threads of different blocks is only achieved through global memory, and they are synchronized by ending a kernel.

All the threads within a block are grouped into *warps*. A warp is a collection of threads that can actually run concurrently (with no time-sharing) on a given multiprocessor. The developer can decide the number of threads to be executed (up to a limit intrinsic to CUDA), but if there are more threads than the warp size, they are executed with time-sharing on the available hardware resources.

In the CUDA model, threads can access the whole GPU global memory, but there is a performance boost when threads access data stored in shared memory, which is explicitly managed. In order to make the most efficient usage of the GPU's computational resources, large data structures are stored in global memory, and the shared memory should be prioritized for storing strategic, often-used data structures. These hardware characteristics can have a big impact for accelerating cardiac electromechanical simulations through GPUs.

## 4. MODEL DESCRIPTIONS AND NUMERICAL METHODS

In this section, we introduce the electrophysiology and mechanical models used for modeling weakly coupled electromechanics. In the case of electrophysiology, we introduce the monodomain model and its solution using second-order Strang splitting (see Section 4.1). This description is followed by an outline of the quasi-static finite elasticity equations applied to cardiac mechanics and its solution using finite elements (see Section 4.2).

### 4.1. Electrophysiology problem

Modeling electrophysiology in the heart is typically accomplished using the monodomain [20, 21] or bidomain [22–26] equations, which simulate the spread of membrane potential or intra/extracellular potential, respectively. In this paper, we focus on modeling the electrophysiology in the heart, denoted by the domain 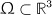 (with boundary δΩ, using the monodomain model). Here, we seek a membrane potential 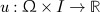 and the *m* − cell model variables 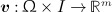 over some time interval *I* = [0,*T*] satisfying [27],


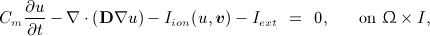
(1)


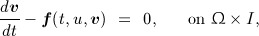
(2)


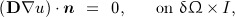
(3)


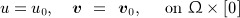
(4)

where 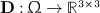 is the diffusion tensor related to the gap junctions between cells and membrane capacitance. *I*_*ion*_(*u*,*v*) is the total ionic current (which is a function of the voltage *u*, the gating variables and ion concentrations), 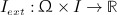 is the stimulus current, ***f*** is a function governing rate-of-change in the *m* − cell model variables, and ***n*** is the normal to the surface of the boundary δΩ. The diffusion tensor **D** is of the form 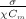, where *σ* is the conductivity, *C*_*m*_ is the membrane capacitance, and *χ* is the cell surface to volume ratio. In this paper, we have defined *σ* using *σ* = *σ*_*i*_*σ*_*e*_(*σ*_*e*_ + *sσ*_*i*_)^ − 1^, where the intra-longitudinal, intra-transversal, extra-longitudinal, and extra-transversal conductivity values are 0.17, 0.019, 0.62, and 0.24 S/m, respectively. In our simulations, the value for membrane capacitance *C*_*m*_ was 0.185  μ*F* and *χ* was 140 *mm*^ − 1^. In this model, an external stimulus current *I*_*ext*_of 35 mV/ms is applied at a time between 0 and 2 ms.

A wide variety of mathematical methods have been applied to solve the monodomain equations, including finite difference methods [28], FEMs [29–31], and finite volume methods [32]. Here, we solve the monodomain equations using the FEM, seeking solutions *u* ∈ *U* and ***v*** ∈ ***s****V*,


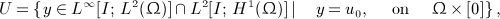



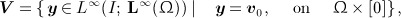


which satisfy the weak formulation of Equations (1)–(4) derived by the standard Galerkin procedure [33], that is,


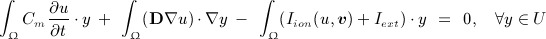
(5)


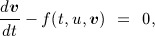
(6)

#### 4.1.1. Discrete electrophysiology problem and solution

**Figure 2:**
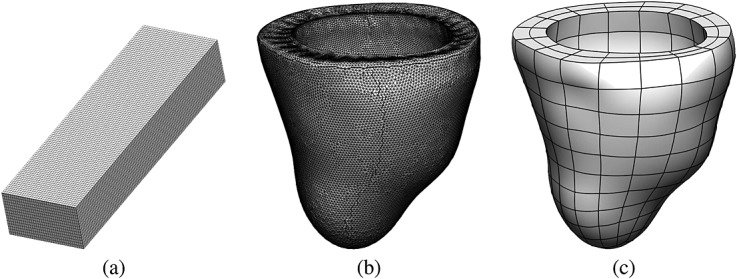
(a) Benchmark problem mesh. For this mesh, we have used the following resolutions: 0.2 mm ( ∼ 58 K DOFs), 0.1 mm ( ∼ 443 K DOFs), and 0.05 mm ( ∼ 3.5 M DOFs); (b) LV mesh. For this mesh, we have used the following resolutions: 0.5 mm ( ∼ 2.5 M DOFs) and the second mesh a resolution of 0.2 mm ( ∼ 19 M DOFs); (c) mechanics mesh—with 352 quadratic hexahedral elements and 555 nodes (3605 DOFs).

In this paper, we focus on the solution of the monodomain problem on tetrahedral and hexahedral grids. Here, an approximation Ω_*h*_ of Ω is constructed by merging finitely many, non-overlapping elements, *τ*, which assemble to form the mesh, *T*_*h*_(Ω) (see Figure 2), that is,


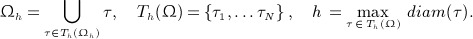


The time domain, *I*, is first divided into *N*_*I*_ non-overlapping intervals (*t*^*n* − 1^,*t*^*n*^), *t*^*n* − 1^ < *t*^*n*^, *t*^0^ = 0 and *t*^*N*^ = *T*, which denote the time stepping sequence for the PDE (Equation (5)). However, as the kinetics of the cell model have characteristic behavior that vary in space and time, the stepping sequence may be further subdivided into *r* substeps, which are applied adaptively in the ODE [34], that is,


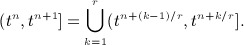


Over each time interval or subinterval, the membrane potential and cell model variables are taken as constants in time, respectively. As a result, the solution to the PDE system at each time step is approximated in


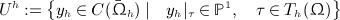


Letting 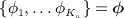 denote the basis of *U*^*h*^ (where *K*_*u*_ =  span *U*^*h*^), each PDE solution step may be expressed as the weighted sum *u*_*h*_ = *U* · ***ϕ***. In general, the approximation of cell model variables, ***v***_*h*_, in the discrete setting may be handled a number of ways. In some cases, cell variables have been approximated at all quadrature points in Ω_*h*_, whereas others approximate cell variables at mesh vertices (see [30] for more details). In either case, the solution at each substep of the ODE system is solved at distinct points ***P*** = {***p***_*k*_}, that is,


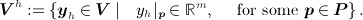


In this case, the ODE model system is then solved independently at each discrete point (letting *V* denote the total vector of ODE state variables), and its values interpolated between points (if necessary). Finally, using a backward Euler discretization of the time derivatives in Equation (5) and adaptive forward Euler in Equation (6), we may pose the discrete finite element weak form system as


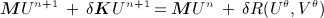
(7)


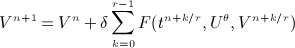
(8)

where


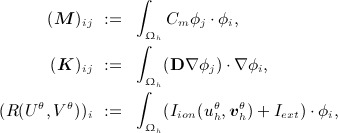


and *θ* ∈ [*n*,*n* + 1]. Note that *θ* = *n* corresponds to a semi-implicit scheme, whereas *θ* = *n* + 1 represents a fully implicit scheme. The vector function *F* denotes the application of ***f*** for each discrete point ***P***, detailing the dynamics of the cell. These cellular dynamics may be described using models such as the Luo Rudy [35] or ten Tusscher and Panfilov 2006 [36]. In this paper, the simulations were performed with the cell model described by the commonly used ten Tusscher and Panfilov 2006 model, resulting in a system of 19 variables at each ***p*** ∈ ***P***. We note that in Equation (8), *r* is selected adaptively both in space (that is, for each ***p*** ∈ ***P***) and time based on the rate of change of membrane potential 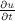 [34]. This allows the numerical solver to take a small time during the fast upstroke of the cardiac action potential and bigger time steps at other times.

An alternative approach—which is followed in this paper—providing improved computational efficiency is so-called Strang splitting for the monodomain problem [37]. The crux of this approach is splitting the discrete operator into linear PDE and nonlinear ODE parts [38] as shown in Equations (9)–(12).


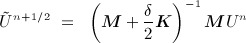
(9)


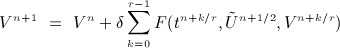
(10)


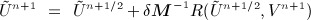
(11)


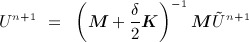
(12)

Note that by choosing the points ***P*** to correspond to nodes of *T*_*h*_(Ω) and approximating *I*_*ion*_ and *I*_*ext*_ linearly over each element, the matrix solve in Equation (11) may be eliminated. This has been shown to improve efficiency while preserving the accuracy of the method subject to reasonable limits on time step [30, 34, 39–41].

### 4.2. Cardiac mechanics problem

The cardiac myocardium is typically modeled as a hyperelastic material and solved using quasi-static finite elasticity theory [2, 15]. The aim of simulating cardiac tissue mechanics is to find a displacement field 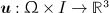 giving the deformed position,


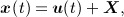


for every point in ***X*** ∈ Ω and time *t* ∈ *I*. In the case of incompressible mixed formulation, we also solve for the hydrostatic pressure 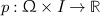, providing the force to constrain volume change. The displacement and pressure are then found by considering the saddle point of the quasi-static Helmholtz potential, 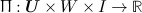 at each *t* ∈ *I*, that is,


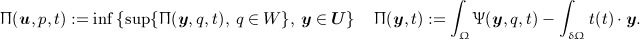


Here, Π represents the balance of internal strain energy (given by Ψ) and the applied external energy. The solution is sought in (***u***,*p*) ∈ *L*^ ∞ ^(*I*;***U***) × *L*^ ∞ ^(*I*,*W*) with


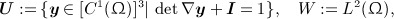


ensuring that deformed body 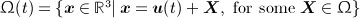 is a well-posed.

The resultant saddle point at each point in time is then sought by finding the zeros of *D*_*u*_Π(***u***,*p*,*t*)(***y***) and *D*_*p*_Π(***u***,*p*,*t*)(*q*). That is, by finding the point in ***U*** × *W* for which the derivative in the direction of any function in the space ***U*** or *W* is zero [42], that is,


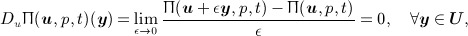
(13)


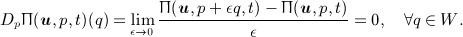
(14)

The internal strain energy, 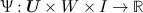, in cardiac mechanics is typically defined in terms of the deformation gradient ***F*** = ∇ ***u*** + ***I***, right Cauchy Green tensor ***C*** = ***F***^*T*^***F*** or Green strain 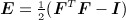 [42–44]. Thus, to apply the directional derivative in Equation (13), we must apply the chain rule. Considering the case where Ψ is a function of the Green strain, the weak form equation may be stated as follows: find (***u***,*p*)(*t*) ∈ ***U*** × *W* such that


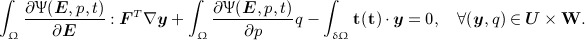
(15)

#### 4.2.1. Cardiac constitutive law and boundary conditions

In this paper, we modeled the myocardium using the anisotropic Costa law [45] combined with the active contraction law in [46, 47]. Anisotropy was modeled using three vectors {*q*_1_,*q*_2_*q*_3_} denoting the fiber, sheet, and sheet normal directions, respectively. Defined on *Ω*, these vectors are mutually orthogonal and of unit length at all points in space, thus forming a basis oriented in the local microstructural directions [48].


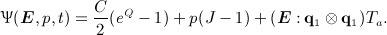
(16)

The first term in Equation (16) details the passive components, where *Q* is defined to be


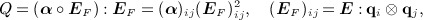
(17)

and ***α*** is symmetric tensor of coefficients, which scale strain with respect to local microstructural directions. The resistance to volume change is provided by the second term of Equation (16), which adds internal energy if *J* − 1 ≠ 0. Last, the active contraction in the tissue was generated using the Niederer contraction model [47]. This six-parameter model captures the length dependent rates of tension development, along with peak tension.‡ In the model, active tension, *T*_*a*_, was defined as


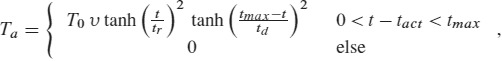
(18)


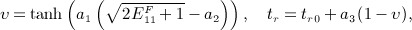
(19)

where *a*_1_ corresponds to the degree of length dependence, *a*_2_ is the length at which no tension is generated, *a*_3_ is a scalar of length dependent activation, *t*_*act*_ is the time of cellular activation computed from the electrophysiology model, *t*_*r*0_ is the baseline activation time constant, *t*_*d*_ is the relaxation time constant, *t*_*max*_ is the duration of tension generation, and *T*_0_ is the peak isometric tension. The function *t*_*r*_ regulates the rise time of the tension transient, whereas *υ* is a nonlinear length dependent function.

Simulating the heart cycle, the heart model was coupled to the Shi Windkessel model [49, 50], representing the dynamic load imposed by the systemic and pulmonary vascular compartments. Coupling was enforced using an additional Lagrange multiplier (scalar multiplied by the unit normal) applied on the endocardial boundary, which was used to impose endocardial volume change. The computed multiplier, denoting pressure, was then passed to the Windkessel model, which was used to compute the associated volume change (within a fixed point iteration).

#### 4.2.2. Discrete mechanics problem and solution

As in Section 4.1, the solution to Equation (15) is approximated using the FEM. Constructing the solid mechanical mesh *S*_*h*_(Ω) (see Figure 2) and approximation spaces,


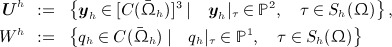


Similarly, the time domain, *I*, is first divided into *N*_*I*_ non-overlapping intervals (*t*^*n* − 1^,*t*^*n*^), *t*^*n* − 1^ < *t*^*n*^, *t*^0^ = 0, and *t*^*N*^ = *T*. Letting 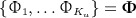 and 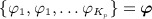 denote the basis of ***U***^*h*^ and *W*^*h*^, respectively, (where *K*_***u***_ =  span ***U***^*h*^ and *K*_*p*_ =  span *W*^*h*^), then the solution at the *n*^*th*^ time step may be written as 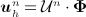 and 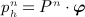. The resulting weak form may then be written as


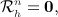
(20)

where


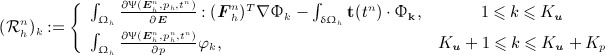
(21)

represents the residual function. The nonlinear mechanics system is subsequently solved using the Newton–Raphson scheme with line search and Jacobian reuse outlined in [51].

## 5. PARALLEL IMPLEMENTATIONS

This section describes both the GPU implementation and the CPU multi-physics software providing the infrastructure into which the GPU code is integrated. CPU and GPU codes have been developed in different languages (FORTRAN2003 and CUDA). Both CPU and GPU parts have been integrated, defining data structures to act as input and output interfaces. These interfaces are updated in the following way. During each simulation cycle, the CPU code updates the input data associated to the GPU functionality (ODE solve, PDE solve or mechanics) before running it. Once the GPU code finishes, output data is copied back to CPU memory, and the CPU side proceeds with the simulation. The following subsections explain different parallelization strategies of CPU and GPU parts.

### 5.1. CPU implementation

All CPU simulations were run in the finite element code 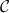
Heart. Developed for modeling multi-physics fluid-structure interaction in the heart [52–56], 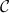
Heart has been further developed to incorporate additional physical systems and provide flexible multi-physics integration. Support for many finite element discretization schemes, physics, and coupling along with domain partition and parallelization are some of the core features of 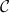
Heart. The automatic domain partition is carried out using the widely available open source software ParMetis [57]. Partitioning is computed in parallel, using an element-based partition, in which each subdomain is uniquely assigned to an individual core. Subsequently, all FEM-based procedures are computed over elements on a core and requisite computations passed between ranks to form global residuals and matrices using MPI. In this way, the original mesh is partitioned by minimizing communication surface between subdomains and maximizing load balance. Parallelization is carried out at a low level, so, if properly coded, each of the coupled problem retains a good scalability. For solving the linear systems resulting from the different multi-physics problems, 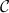
Heart uses a number of established libraries including PETSc [58], MUMPS [59], and SuperLU [60]. For the monodomain problem, the algebraic system of equations is solved using Jacobi-preconditioned CG within PETSc. For the mechanics problem, MUMPS (a direct parallel solver) was used for solving the system of PDEs. Although direct methods are known to exhibit suboptimal scalability (with system size as well as the number of cores), they are particularly efficient for the mechanical system considered in this study.

### 5.2. GPU implementation

#### 5.2.1. Cardiac electrical activation

We have implemented on the GPU the solution process to Equations (1) and (2) of the electrical activation problem, as two different parts. The first part performs the solution of the system of ODEs, and the second one performs the solution of the system of PDEs present in the monodomain equation.

The integration of the state variables of each cell model is a trivially parallel task ideally suited for SIMD processing. This is due to the decoupling of each ODE model in space, which involves an update of each state variable that has no implicit dependence on membrane potential or implicit/explicit dependence on state variables at other nodes. For this reason, each GPU thread updates the cell model state variables in parallel and integrates the computed values. To further capitalize on the GPU architecture, the ODE model was coded to use fast on-chip memory, loading cell model parameters onto the shared memory of each GPU block. In this way, threads within the same block can share these values, reducing the memory latency.

Another factor that limits the GPU performance is the per thread register bench usage. The use of this resource is based on the private (local) data and number of instructions in a GPU kernel (function). The number of state variables of the implemented cell model and the set of equations involved results in high register usage values. For this reason, a number of automatic transformations are performed within the cell model code. The initial C language code was obtained from the publicly available repository CellML [61]. This code was then automatically transformed by reducing the number of temporal variables required. In addition, operations in the cell model equations involving constant values were evaluated and collapsed to a single value. These transformations allowed us to obtain a more efficient usage of the private register bench of each GPU thread.

Performance of the ODE code was further improved by reducing the GPU thread divergence. On the GPU, as opposed to the CPU case, threads within a block run concurrently only if they execute the same instruction. However, threads might follow different branches in a conditional statement, reducing the synchronization between threads and thus reducing the parallelism. To avoid this issue with conditionals (which are often used within cell models), we instead employ Heaviside functions. In this way, we can mimic the conditional as a product between a literal and the condition of the Heaviside function. In this way, results of the function are calculated, whereas conditional statements are avoided.

The other part of the monodomain problem ported to the GPU is the solution step for the system of PDEs. We have implemented the Jacobi preconditioned conjugate gradient (CG) method [62]. For the PDE implementation, a hybrid approach has been adopted. In this hybrid version, the CPU controls the code flow of the CG method (i.e., evaluates conditions such as termination criteria, etc.), and the GPU performs in parallel vector–vector operations present in the CG method. In order to optimize productivity while maintaining the efficiency of the GPU code, vector–vector operations have been implemented using two CUDA libraries (CUSPARSE§ and CUBLAS¶ ). At each EP simulation step, the system of PDEs is solved by copying the required data from CPU memory to GPU memory, the system is then solved using the GPU-based Jacobi–CG implementation and the result copied back to CPU memory.

#### 5.2.2. Cardiac mechanics

To accelerate the simulation of cardiac mechanics, we examined the first-order effects, which influence the compute time of the whole cycle. Because of the system matrix reuse strategy which significantly improves compute times [51] by reducing the number of matrix builds and factorizations, residual evaluations consume most of the compute time. As a result, our initial focus was to port both residual evaluations and Jacobian computations to the GPU. The Jacobian was computed and the residual evaluated locally for each element and later added to both the global Jacobian and residual. These per element Jacobian and residual computations have been parallelized on the GPU.

There are some calculations common to both the Jacobian computation and the residual evaluation. Specifically, these are related to the computation of the tensors and terms involved in the mechanics equations as well as the stress computation according to the constitutive law. Because each of these terms are evaluated at gauss points, their computation has also been parallelized on the GPU by assigning each gauss point computations to a GPU block. In this way, tensor operations for each gauss point are executed in parallel by threads within a GPU block.

Using the different mechanics term computations, the Jacobian of each element is calculated by means of a central finite difference method, which perturbs the displacement solution and re-evaluates all the mechanics terms and the residual. This perturbation method iterates for each element over the number of nodes, *n*, and each dimension of the displacement variable, *d*. Thus, the number of iterations is *n* * *d*. The central perturbation method has been parallelized by assigning to each GPU block computations of each dimension at each node (i.e., the number of blocks launched is *n* * *d*).

After the Jacobian is computed, the residual is then evaluated at each node for both the displacement and pressure variables. For this reason, this task has been parallelized by assigning to each GPU block computations of each node (i.e., the number of blocks launched is equal to the number of displacement nodes plus number of pressure nodes).

## 6. RESULTS

This section reports on performance improvements provided by the GPU for both the electrical and mechanical components of the cardiac simulations and the impact, in terms of execution times, that such improvements can have within the clinical context. Regarding the electrophysiology problem, we have run a range of different monodomain simulations with different mesh sizes. In order to check the performance when the mesh resolution decreases, we have used a recently established benchmark for the simulation of electrical activation [47]. To be consistent with the previous benchmark study, we have used a PDE time step of 0.01 ms and an ODE time step of 0.0005 ms for different resolutions (see Figure 2(a)); field variables within all of these meshes were interpolated using linear basis functions.

We have also obtained results using a realistic mesh of the human left ventricle (LV) at two different resolutions (see Figure 2(b)), both meshes again used linear basis functions. We have obtained activation time values by simulating 300 ms of electrical activity, setting the PDE time step to 0.01 ms and ODE time step to 0.005 ms. Adaptive stepping [34] allows us to use this small step size of 0.005 ms only during upstroke and alter the ODE step to a higher step size of 1/33 ms rest of the time. For the finest resolution meshes, these time step values were shown previously to be sufficient for numerical convergence [47]. Electrical activation was solved on the deformed mesh at end-diastole, reflecting the geometry at which activation typically occurs. Further, as the generation of contraction in the normal heart typically occurs on a longer time scale and the physiological significance of deformation on electrical conductivity remains debated, the mesh was assumed static.

**Figure 3:**
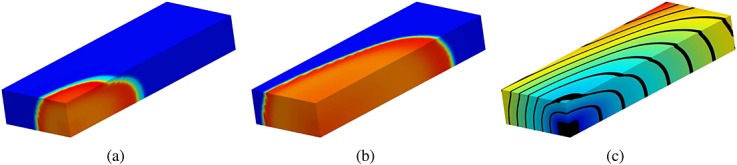
(a) and (b), Electrical activity propagation in the benchmark mesh at different simulation stages represented by a color map from dark blue ( − 86) to red (35); (c) activation times represented by a color map from dark blue (0) to red (57) and contour bands.

**Figure 4:**
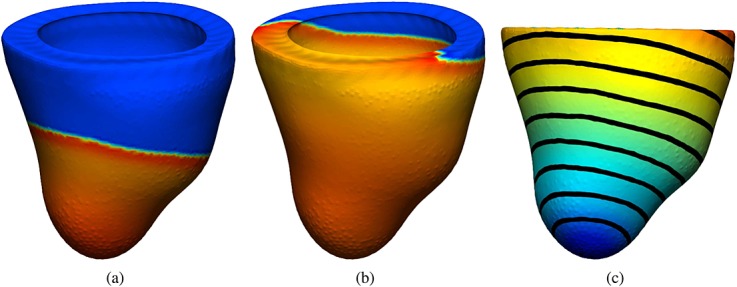
(a) and (b) Electrical activity propagation in a human left ventricular mesh at different simulation stages represented by a color map from dark blue ( − 86) to red (35); (c) activation times represented by a color map from dark blue (0) to red (75) and contour bands.

Figure 3(a,b) shows the propagation of the membrane potential in the benchmark mesh with resolution 0.2 mm at two different stages when using the GPU. Figure 3 shows the activation times for the same resolution, represented by a color map and contour bands. Figure 4(a,b) shows the propagation of the membrane potential in the human left ventricular mesh at different stages when using the GPU. Figure 4 shows the activation times for the same mesh, represented by a color map and contour bands.

**Figure 5:**
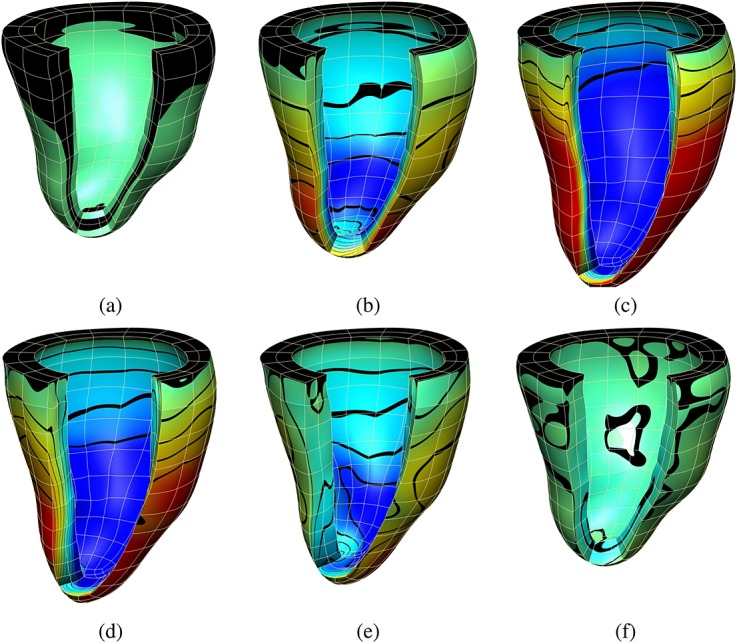
(a), (b), and (c) show the displacement values in the fiber direction at three time steps during diastole; (d), (e), and (f) at three time steps during systole. Displacement values are represented by a color map from dark blue ( − 5.0) to red (5.9) and contour bands.

**Figure 6:**
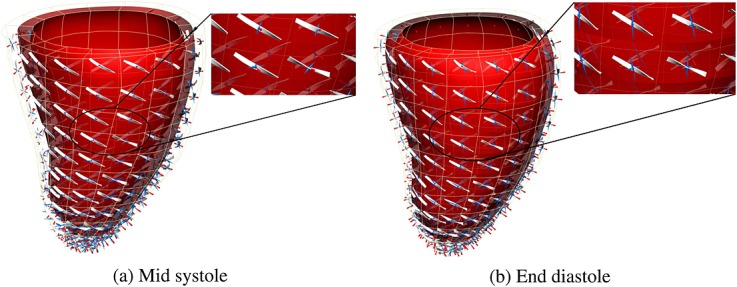
Detail of fibers and strain tensor (a) at mid systole (b) at end of diastole. Glyphs represent principle components of strain as follows: blue (stretch) and red (compression).

For mechanics problem, we have simulated the model described in Section 4.2, and we have used the same LV human geometry as for the electrophysiology problem. However, in this case, it has been discretized on the basis of a coarser mesh (see Figure 2(c)), reflecting the type of meshes often observed in cardiac mechanics (although the results illustrated are expected to be consistent with larger cardiac mechanics meshes). To solve the mechanics problem on this mesh, we have mapped the activation time from the fine grid electrophysiology mesh onto the mechanics mesh. Using these activation time values, we simulated the cardiac cycle, comparing performance over a single beat (with a duration of 1 s and a time step of 0.001 ms). Figure 5 shows displacement values during a cycle simulation. Figure 6 shows the principal strain vectors and fibers at end diastole and mid systole steps.

The different implementations have been compared using GPU, SC CPU, and MC CPU platform configurations. We enumerate the specifications of the different platforms in order to do a performance/price/power consumption comparison. For the CPU simulations, we used a machine with 32-core AMD Opterons @ 2.0 GHz and 128 GB of RAM shared among all cores. The processor used in the CPU tests has a power consumption range of ∼ 800/520 W and a price of ∼ £4100. The theoretical performance of this platform per core is 17.75 GFlops. For our GPU simulations, we have used up to four Tesla C2070 processors, each one with 448 SPs and 6 GB of device memory. The theoretical performance of the GPU processor is 515 GFlops, with a power consumption of 238 W and a price of ∼ £1600. Previous works comparing CPU/GPU implementations of biomedical problems do a single core CPU/single GPU comparison without taking into account the theoretical performance of each architecture. For this reason, we compare the performance of the GPU implementation executed on a Tesla C2070 processor with up to 32 CPU cores (with a theoretical peak performance of 568 GFlops). In this way, we are able to analyze the performance provided by the single core, multi-core, and GPU implementations. The accuracy of GPU implementations has been determined by comparing results provided by GPU and CPU simulations. For the monodomain problem, we compared membrane potential values obtaining a maximum difference of 1.0E-13. For the mechanics problem, we compared displacement, pressure, and fiber field values obtaining a maximum difference of 1.0E-15.

**Figure 7:**
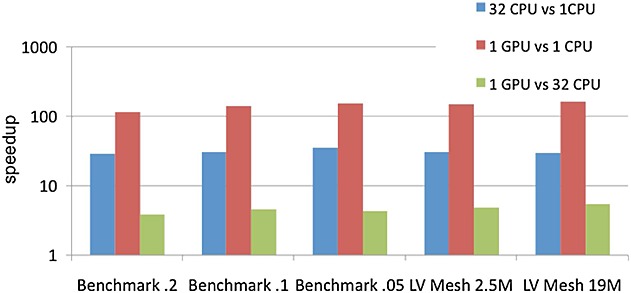
Speedup obtained by the different parallel platforms for the ODE problem.

Figure 7 shows the speedup obtained by the different parallel platforms when solving the ODE problem. These results clearly demonstrate that the GPU outperforms both the sequential and parallel CPU versions. They also demonstrate that the performance is further improved when the problem size increases mainly because of the high memory bandwidth available on the GPU. On the other hand, Figure 7 shows that the GPU version is always faster than the multi-core CPU despite the fact that the CPU platform has a slightly higher theoretical performance. Specifically, the GPU achieves a 5.5 × speedup compared with the MC CPU for the biggest LV mesh with respect to the multi-core CPU. This speedup allows to reduce the ODE run time from 93 h when using the MC CPU down to 17 h when using the GPU for the largest LV mesh.

**Figure 8:**
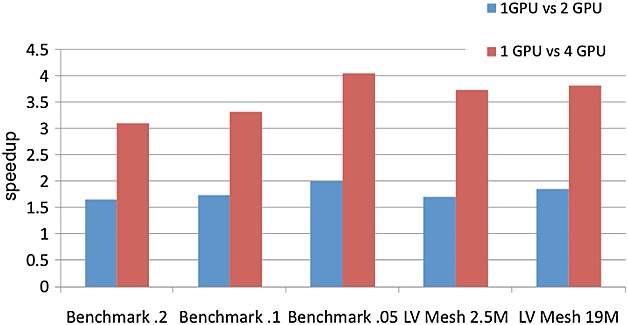
Scalability of the ODE problem when using multiple GPUs.

Figure 8 shows the speedup obtained when comparing a single GPU version with respect to two and four GPUs, demonstrating the scalability of the ODE problem when using several GPUs. Results show that almost linear scalability is obtained for the ODE problem. Furthermore, when comparing the results grouped by mesh type, it can be seen that the performance is further increased with the problem size evidenced by the acceleration for the bigger LV mesh which is higher in comparison with the smaller LV mesh. These results demonstrate the potential of the GPU architecture for accelerating the ODE component of the solution procedure, specially for large-scale geometries.

**Figure 9:**
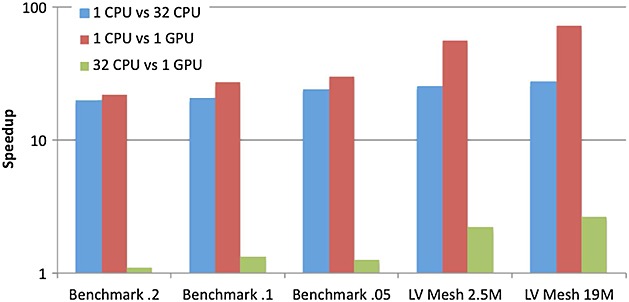
Speedup obtained by the different parallel platforms for the PDE problem.

Figure 9 shows the speedup obtained by the different parallel platforms considered when solving the PDE problem. Speedup values for the CPU versions have been obtained using the execution times provided by PETSc, which is the library used for solving the PDE. This comparison again demonstrates that the GPU outperforms both the SC and MC CPU versions. Performance is also further improved when the problem size increases. Unlike the ODE problem, the PDE algorithm provides lower acceleration rates between the GPU and the different CPU platforms. This is mainly because the CG algorithm requires more synchronization among GPU threads, resulting in a performance degradation. Nevertheless, the GPU version always outperforms the MC CPU achieving up to 2.6 × speedup for the largest LV mesh. This speedup allows to reduce the PDE run time from 53 h when using the MC CPU down to 20 h when using the GPU for the largest LV mesh.

Tables I and II show the performance provided by the different platforms when running a whole cycle mechanics simulation. Table I shows the total run time (in seconds) for the GPU and CPU versions and the percentage of the total run time that the three main tasks of the simulation take (i.e., Jacobian computation, residual evaluation, and PDE solve). We report results for these three tasks because for the single CPU version, they take most of the simulation run time (28146.5 s). When the mechanics simulation is parallelized on the multi-core CPU, the total run time is significantly reduced (1481.4 s). For the 32-core CPU version, the percentage of the total run time required by the Jacobian computation and the residual evaluation are reduced, but for the PDE solution, the step is increased (10%), meaning that the latter task provides a lower scalability. Looking at the GPU results, the percentage of time required by the Jacobian and residual computations is further decreased.

**Table I:** Total run time split in mechanics cycle simulation.

Stage	1_CPU_	32_CPU_	1_GPU_
Jacobian	19%	15%	8%
Residual	79%	73%	43%
PDE solve	2%	10%	39%
Total time	28146.5 s	1481.4 s	1481.6 s

**Table II:** Speedup of the parallel CPU and GPU implementations with respect to the sequential CPU version.

Stage	1_CPU_/32_CPU_	1_CPU_/1_GPU_	32_CPU_/1_GPU_
Jacobian	22.3 ×	44.5 ×	2.0 ×
Residual	20.4 ×	34.8 ×	1.7 ×
PDE solve	3.7 ×	1.0 ×	0.3 ×

For heart problem, the MC CPU required 1585 s for 1 ms of simulated time on the largest LV mesh, whereas the GPU required only 400 s, resulting in a significant improvement in computation time. The performance was nearly equivalent for the solid mechanics problem, with both the MC CPU/1 GPU requiring 1.5 s for 1 ms of simulated time. However, this is due to the non-parallelized PDE solve, which is 3.7 × faster in the MC CPU simulation (see Table II). Accounting for this time, we see that significant improvement in the MC CPU computation time.

Table II shows the speedup of the parallel CPU and GPU versions with respect to the sequential CPU version as well as the speedup of the GPU with respect to the parallel CPU version. This speedup evaluation between the different platforms has been performed by comparing the execution time of the three tasks of the simulation: the PDE solve time and the two parts of mechanics implemented on the GPU (i.e., Jacobian computation and residual evaluation). Looking at these results, it can be seen that tasks implemented on the GPU outperform both the sequential and parallel CPU versions of the same tasks. Comparing the sequential CPU and GPU versions, the following acceleration factors are obtained: 44.5 × (Jacobian computation) and 34.8 × (residual evaluation). When comparing the parallel CPU and GPU versions, the following acceleration factors are obtained: 2.0 × (Jacobian computation) and 1.7 × (residual evaluation). However, the PDE solve task only runs in parallel for the 32-core version and runs sequentially for the single CPU and GPU simulations. The acceleration factors provided by the parallel CPU and GPU versions enable the simulation of one cycle in around 25 min. However, if we consider the same PDE solve time for the GPU simulation as for the 32-core simulation, the GPU total run time is decreased to 15 min. It should be noticed that the GPU can improve the computational performance of electromechanical simulations while the price ratio MC CPU/GPU is around 2.5 (i.e., the GPU is 2.5 × cheaper) and the power consumption ratio MC CPU/GPU is 3.36–2.18 (i.e., the GPU consumes less energy).

## 7. CONCLUSIONS

The application of electromechanical models within time sensitive environments such as the clinic, requires significant advancement of the computational software used to solve both cardiac electrical activation and mechanics. Previous efforts have addressed this problem by efficiently exploiting the computational capabilities of HPC based on clusters of CPU processors. Although significant speedups were obtained, these platforms have the disadvantage of a high cost in terms of price and power consumption. For tackling these problems, the GPU has arisen as an efficient platform providing a good power/performance ratio. Previous works have proposed the use of GPUs for solving the cardiac electrical activation problem. Building on these works, we have shown the potential utility of GPUs for simulating both electrical activation and mechanics within the human heart.

Specifically, we have developed a GPU-based scheme to enable the acceleration of a human scale electrical activation problem and a novel implementation of cardiac mechanics on the GPU. To evaluate the effectiveness of our implementation, we have focused on performing a comparison between a GPU and a multi-core CPU with similar theoretical performance. The GPU implementations were developed to take advantage of the features of this parallel platform and allowed to significantly accelerate the different problems simulated for human scale models. Concretely, for the human LV mesh ( ∼ 19 M. DOFs) speedups of 5.5 × and 2.6 × were achieved for the ODE solve and PDE solution steps, respectively. Regarding mechanics, for the same human geometry, speedups of 1.7 × and 2.0 × were obtained for the residual evaluation time and Jacobian computation time, respectively. The fact that this performance comparison was performed using a GPU and a multi-core CPU with similar theoretical performance provides an unbiased assessment of the capacity for the GPU platform to accelerate computations focused on electromechanical coupling. In addition, the GPU is more efficient offering a price ratio MC CPU/GPU of 2.5 (i.e., the GPU is 2.5 × cheaper) and a power consumption ratio MC CPU/GPU of 3.36–2.18 (i.e., the GPU requires less energy).

Although the results presented in this paper show the benefits that the GPU architecture can provide to simulate VPH cardiac models, some improvements and extensions in functionality are left as future work. In this study, the GPU electrophysiology implementation only uses one cell model. The development of additional cell models for the GPU requires significant technical skill in comparison with coding the same model for the CPU. In order to generalize the use of the GPU platform to the VPH community, it would thus be desirable to develop a tool for automatically generating GPU code and add this functionality to existent cell model repositories [61]. Furthermore, this study proposes the implementation of the PDE solution step in the electrophysiology problem on a single GPU. Some previous studies have solved the system of PDEs using multiple GPUs [9]. In this approach, the communication between GPUs is handled by the CPU through MPI. However, CUDA has recently released a new peer-to-peer communication method where GPUs, within the same node, communicate directly through the Peripheral Component Interconnect (PCI) bus. In this way, a hierarchical method could be adopted where GPUs hosted in different nodes communicate through MPI, and GPUs within the same node communicate using the PCI bus interface.

Although the GPU mechanics implementation presented in this study provides a performance improvement with respect to MC CPU, there remains significant further potential for exploiting the GPU capabilities. In the GPU mechanics code, Jacobian and residual per element computations are performed in parallel on the GPU. Nevertheless, the mechanics code could be further accelerated by performing multiple elements computations in parallel. Computing multiple elements in parallel results in a higher consumption of GPU memory. However, the acceleration obtained justifies the higher memory required. This has been already observed in our mechanics implementation for the Jacobian computations. The Jacobian matrix is built using a perturbation method, which iterates over the number of DOFs of the mechanics problem. Because this loop is parallelized on the GPU, a higher speedup factor is provided for the Jacobian computation (2.0 × ) with respect to the residual evaluation (1.7 × ). Furthermore, because mechanics run time is dominated by residual computations (see Table I), the reduction of residual time should result in a significant acceleration of the total run time. As presented in the results section, the PDE solve step in the mechanics simulation was run sequentially on the CPU. This step can be also parallelized implementing on the GPU a direct solver or a preconditioned iterative solver. This parallelization of the PDE solve step would lead to a reduction of the mechanics total time.

## Notes

‡ Note that following [46, 47] the length dependence of *T*_*a*_ is not considered when taking *∂*/*∂****E***.

§ http://developer.nvidia.com/cuda/cusparse

¶ http://developer.nvidia.com/cuda/cublas
